# Soluble MFGE8 mediates cell entry of Crimean-Congo hemorrhagic fever virus

**DOI:** 10.1128/mbio.01617-25

**Published:** 2025-08-25

**Authors:** Xue Ma, Zhi-Sheng Xu, Yan Fu, Yanlong Ma, Wen-Tian Du, Qian Li, Ran Zhan, Sicheng Tian, Lulu Yang, Ziqiao Wang, Fei Feng, Zhichao Gao, Manli Wang, Sheng Cao, Yan-Yi Wang, Rong Zhang

**Affiliations:** 1 Key Laboratory of Medical Molecular Virology (MOE/NHC/CAMS), Shanghai Institute of Infectious Disease and Biosecurity, Shanghai Frontiers Science Center of Pathogenic Microorganisms and Infection, School of Basic Medical Sciences, Shanghai Medical College, Fudan University 58305, Shanghai, China; 2 Key Laboratory of Virology and Biosafety, State Key Laboratory of Virology, Wuhan Institute of Virology, Chinese Academy of Sciences 74614 , Wuhan, China; Virginia Tech, Blacksburg, Virginia, USA

**Keywords:** Crimean-Congo hemorrhagic fever virus, virus entry, MFGE8

## Abstract

Crimean Congo hemorrhagic fever virus (CCHFV) causes fatal tick-borne disease in humans and is a priority pathogen of the World Health Organization. No licensed vaccines or specific antiviral drugs are available. To understand the cell entry of CCHFV and identify potential antiviral targets to combat the disease, here, we perform the CRISPR knockout screen in wild-type cells, followed by a complementary CRISPR activation screen in cells deficient in common attachment factors (heparan sulfate, AXL, TIM-1). We identify the soluble milk fat globule-EGF factor 8 protein (MFGE8), also known as lactadherin, as a proviral factor for CCHFV infection. Overexpression of MFGE8 enhances the pseudotyped, tecVLP, and authentic CCHFV infection, while knockout decreases the infection. MFGE8 is found to promote the virus binding and internalization. Expression of MFGE8 with D48E mutation of the RGD motif and the use of pharmacological inhibitor and gene-editing suggests that MFGE8 mediates virus entry through integrin receptors on the cell surface. Further study demonstrates that soluble MFGE8 protein acts as a bridge to support the entry by binding to not only the reported phosphatidylserine (PtdSer) but also Gc protein on viral envelope and to integrins on cells. The finding of MFGE8 that can bind directly to Gc protein and the entry mode of CCHFV that employs a soluble protein may expand the tissue tropism and increase the pathogenicity of CCHFV. Our study also provides new insight into the underlying mechanisms of cell entry and development of countermeasures for CCHFV.

**IMPORTANCE:**

CCHFV causes severe hemorrhagic fever outbreaks, with a mortality rate of up to 40%. Countries generally list CCHFV as one of the pathogens that requires the highest biosafety level 4 (BSL-4) of containment, which hinders the study of its cell biology and pathogenesis. LDLR was recently identified as a receptor for CCHFV, but other receptors or co-factors remain to be explored. We perform genome-wide CRISPR screens using a safe replication-competent CCHFV pseudovirus and identify a secretory MFGE8 protein that functions as an entry mediator by binding to both the Gc protein and PtdSer on the viral envelope and to the integrins on the cells. Cell entry mediated by a soluble protein may greatly expand the tissue tropism, and the strategies developed to disturb the interaction of MFGE8 with virions or with integrins may help to mitigate the fatal disease induced by CCHFV.

## INTRODUCTION

Crimean-Congo hemorrhagic fever virus (CCHFV) is a tick-borne virus causing severe disease with a wide geographical distribution and a mortality rate of 30% or higher (1–3). Cases caused by CCHFV infection have been reported in Africa, the Middle East, Asia, as well as Southern and Eastern Europe (4). CCHFV can effectively infect various wild animals, such as small rodents, rabbits, ostriches, water buffaloes, etc. Importantly, humans can also be infected by contact with livestock that do not have obvious diseases (5). The US Food and Drug Administration (FDA) has not yet approved any vaccines or treatments for CCHFV (6).

CCHFV is an enveloped negative sense RNA virus belonging to the *Orthonairovirus* genus in the *Nairoviridae* family of *Bunyavirales* order (7). CCHFV has three genome parts, including small segment (S), medium segment (M), and large segment (L). Fragments S, M, and L encode nucleoprotein (NP), glycoprotein precursor (GPC), and RNA-dependent RNA polymerase (RdRp), respectively (8, 9). The GPC is proteolytically processed to produce two main precursor proteins, 140 kDa PreGn and 85 kDa PreGc, which respectively produce two mature structural proteins Gn (37 kDa) and Gc (75 kDa)(10). Gn and Gc participate in receptor binding and entry (11).

Identifying entry receptors or related co-factors of CCHFV is of significance to understand its tissue and species tropism, and pathogenesis, and help to develop strategies for disease control and prevention. Previous studies reported that nucleolin and DC-SIGN are entry factors for CCHFV, but further experiments are needed to confirm (12, 13). The latest research has found that low-density lipoprotein receptor (LDLR) is an entry factor of CCHFV (14–16). The Gc protein of CCHFV is shown to bind directly to LDLR, which can mediate viral entry into various cell types and play roles in infection and pathogenesis in mice (14, 15). In addition, the apolipoprotein E (ApoE) is found to be incorporated into CCHFV particles and enhances its infectivity by promoting LDLR dependent entry (15, 16). However, the use of LDLR blocking antibodies or soluble LDLR protein could only partially block the CCHFV infection. Similarly, genetic knockout of LDLR in cell lines or mice does not completely prevent viral infection. These results suggest that CCHFV may have other receptors or co-factors that mediate the entry in certain cell types.

To comprehensively uncover host factors that are required for CCHFV entry, we performed genome-wide CRISPR screens using replication-competent vesicular stomatitis virus-based CCHFV pseudovirus (rVSV-CCHFV) carrying the CCHFV GPC (17). From our initial CRISPR knockout screen, we identified a suite of genes that are involved in biosynthesis of heparan sulfate (HS), and some other genes, e.g., AXL and HAVCR1 (TIM-1), which are common viral attachment factors. We then conducted CRISPR activation screen in cells for which those common attachment factors are depleted and identified the milk fat globule-EGF factor 8 protein (MFGE8) that, upon upregulation, significantly promotes pseudotyped, transcription- and entry-competent virus-like particle (tecVLP), and authentic CCHFV infection. Mechanically, the secretory MFGE8 protein mediates the virus entry through integrin receptors on the cell membrane, and MFGE8 can directly bind to not only the reported phosphatidylserine (PtSer) but also Gc protein on virions. The identification of a soluble protein mediating cell entry may significantly expand the tissue tropism and increase the pathogenicity of CCHFV which is notorious for its fatal hemorrhagic systemic disease.

## RESULTS

### CRISPR knockout screen identifies common host factors required for rVSV-CCHFV pseudovirus infection

**Fig 1:**
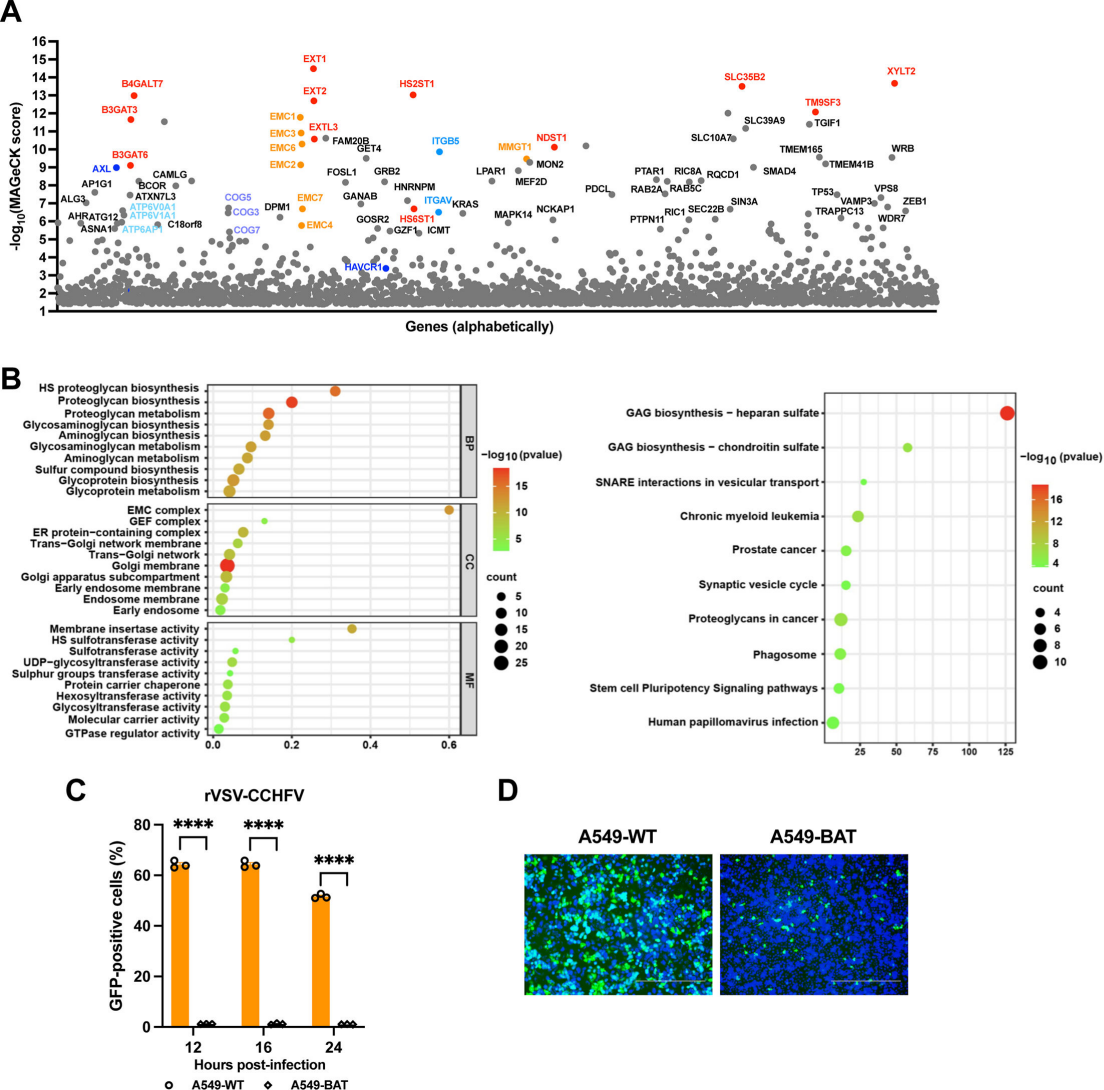
CRISPR knockout screen identifies common host factors required for rVSV-CCHFV pseudovirus infection. (**A**). Bubble plot of genes significantly enriched in a genome-wide CRISPR knockout screen in wild-type A549 (A549-WT) cells challenged with rVSV-CCHFV pseudovirus. The virus-resistant A549-WT cells were collected for analysis, and genes were ranked according to the MAGeCK score. (**B**) KEGG (Kyoto Encyclopedia of Genes and Genomes) and Go (Gene Ontology) analysis of top 100 enriched genes. (**C and D**) Flow cytometry (**C**) and fluorescence imaging (**D**) analysis of A549-WT and A549-BAT (B3GAT3, AXL, and TIM-1 triple-knockout cells) infected with rVSV-CCHFV (MOI 3). The percentage of GFP-positive cells was analyzed at indicated time points using flow cytometer, and images were taken using fluorescence microscope at 24 h post-infection (hpi). Scale bar, 400 µm. Two-way ANOVA with Sidak’s multiple-comparison test. ****P < 0.0001.

In previous work, we demonstrated that heparan sulfate plays a significant role in the adhesion of pseudotyped rVSV-CCHFV bearing the GPC to the plasma membrane of cells (17). To comprehensively uncover host factors required for CCHFV entry, we performed genome-wide CRISPR knockout screen using rVSV-CCHFV as model virus. The viral-resistant cells were harvested for deep sequencing and data analysis (Table S1). As expected, a suite of genes related to heparan sulfate biosynthesis, such as B3GAT3, B4GALT7, EXT1, EXT2, XYLT2, were enriched (Fig. 1A). The genes associated with ER membrane protein complex (EMC1, EMC2, EMC3, etc.), members of V-ATPases (ATP60A1, ATP61A1, etc.), components of the conserved oligomeric Golgi (COG) complex (COG3, COG5, etc.), were also identified (Fig. 1A). In addition, the TAM receptor AXL and TIM receptor HAVCR1 (TIM-1), albeit with low score, showed up. Additionally, ITGAV and ITGB5, encoding the integrins that often form as αVβ5 heterodimer, were identified (Fig. 1A). KEGG and GO analysis also showed the obvious enrichment of genes in the HS proteoglycan biosynthesis pathway (Fig. 1B), suggesting its critical role during rVSV-CCHFV infection.

The first step for viruses to invade cells is to adhere to the cell surface. Many adhesion molecules are involved in this process. Heparan sulfate has been identified as a common attachment factor for many viruses, such as flaviviruses (18), alphaviruses (19), filoviruses (3), and bunyaviruses like Rift Valley fever virus (RVFV) (20). AXL also plays an important role in Dengue virus (21), Zika virus (ZIKV) (22, 23), and SARS-CoV-2 infections (24). As to TIM-1, it has been reported to mediate the infections of Ebola virus, DENV, West Nile virus (25), and hepatitis E virus (HEV)(26). Given the common function of these genes for viral adhesion and entry, we generated A549 triple-knockout cell line that B3GAT3, AXL, and TIM-1 were all depleted (A549-BAT), to examine the effect on rVSV-CCHFV infection. Surprisingly, as compared to wild-type A549 cells (A549-WT), A549-BAT showed a nearly complete resistance to viral infection at 12, 16, and 24 h (Fig. 1C). A549-WT was highly susceptible to rVSV-CCHFV, with the GFP-positive cell rate of up to 70% at 16 h post-infection, while A549-BAT remained largely uninfected at 12, 16, and 24 h, with only 2% of infected-cells at 24 h (Fig. 1C). The representative fluorescent images also showed the marked decrease of infection efficiency in A549-BAT cells for rVSV-CCHFV (Fig. 1D). These results indicated that the infection of rVSV-CCHFV is highly dependent on these common adhesion factors.

### CRISPR activation screen identifies MFGE8 as a proviral host factor for rVSV-CCHFV infection

**Fig 2:**
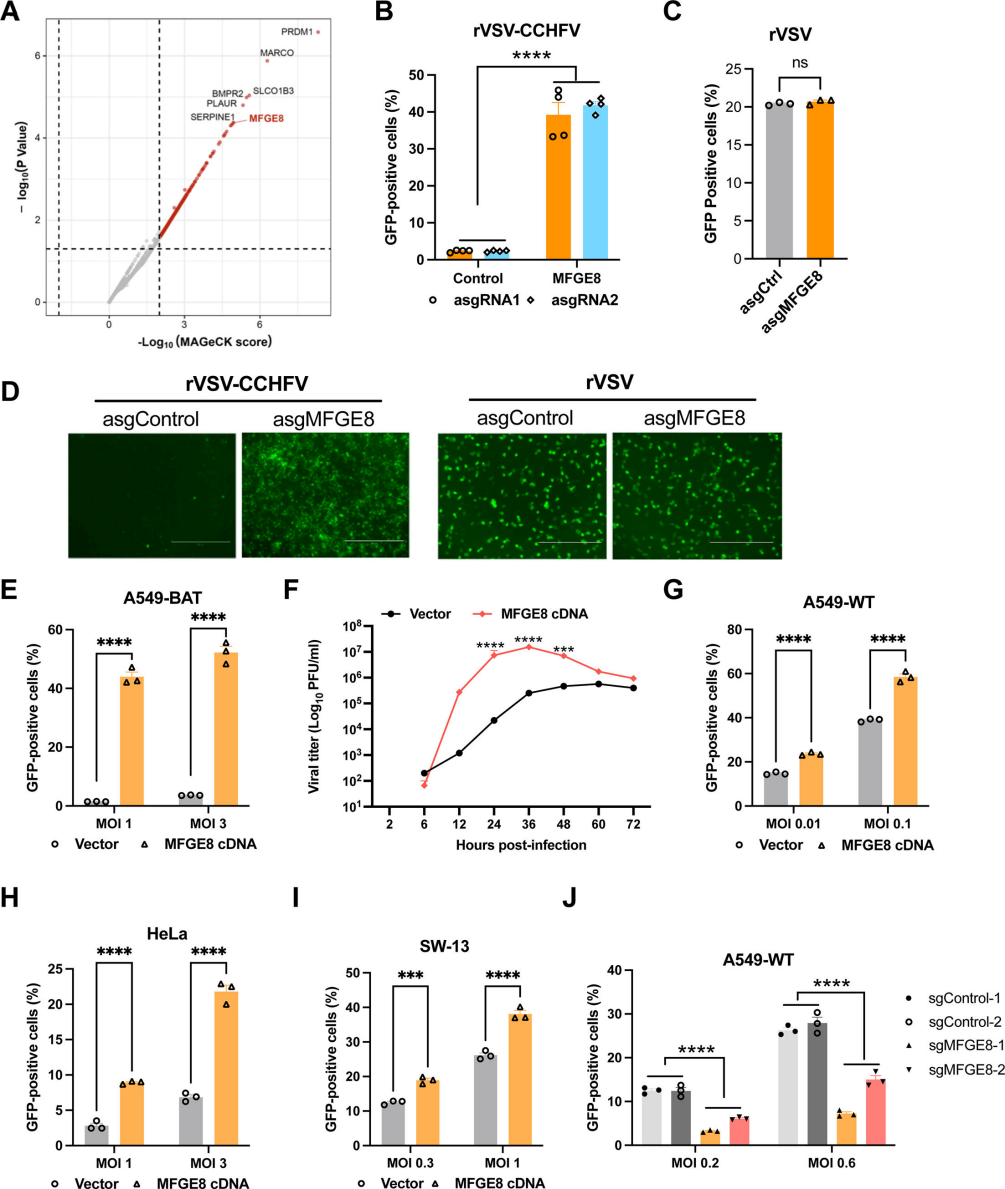
CRISPR activation screen identifies MFGE8 as a proviral host factor for rVSV-CCHFV infection. (**A**) Identification of genes from CRISPR screen in A549-BAT cells. Cells transduced with the CRISPR activation library were infected with rVSV-CCHFV for 24 h. GFP-positive cells were sorted for sgRNA abundance analysis and ranked based on the MAGeCK score and *P* value. (**B and C**) Validation of *MFGE8* gene. Gene expression was activated using two or representative sgRNAs in A549-BAT cells, followed by infection with rVSV-CCHFV (MOI 3, 18 h) (**B**) and rVSV (MOI 0.01, 15 h) (**C**). The percentage of GFP-positive cells were analyzed by flow cytometry. (**D**) Representative fluorescence images of A549-BAT cell infected with respective virus from (**B**) and (**C**) were taken before harvesting the cells. Scale bar, 400 µm. (**E**) Overexpression of MFGE8 enhances rVSV-CCHFV infection in A549-BAT cells. (**F**) Growth kinetics of rVSV-CCHFV in vector control and MFGE8-overexpressing cells. Cells were infected with rVSV-CCHFV at an MOI of 0.3, and viral titers in the supernatants at indicated time points were determined by plaque-forming assay. (**G–I**) Overexpression of MFGE8 enhances rVSV-CCHFV infection in A549-WT (**G**), Hela (**H**), and SW-13 (**I**) cells. The percentage of GFP-positive cells were analyzed by flow cytometry at 16 hpi. (J) Knockout of MFGE8 decreases the rVSV-CCHFV infection. A549-WT cells edited with two different nontargeting control or *MFGE8*-specific sgRNAs were infected with rVSV-CCHFV, followed by flow cytometry analysis of GFP-positive cells at 16 hpi. Two-way ANOVA with Sidak’s multiple-comparison test. ns, not significant; ***P < 0.001; ****P < 0.0001.

To identify other potential entry factors of CCHFV independent of common adhesion molecules such as heparan sulfate as described above, we performed a complementary genome-wide CRISPR activation screen in A549-BAT cells. The cell library was infected with rVSV-CCHFV, and GFP-positive cells were sorted for subsequent extraction of genomic DNA, sgRNA sequencing, and data analysis (Table S1). As shown in Fig. 2A, the top hits from the screen were determined according to their MAGeCK scores and *P* values. After extensive validation with CRISPR activation sgRNAs in A549-BAT cells, we found that MFGE8 showed around 20-fold increase of infection for rVSV-CCHFV, but not rVSV, as determined by flow cytometry and microscopy (Fig. 2B through D). The upregulation of endogenous MFGE8 expression by CRISPR activation sgRNAs was also validated by Western blotting analysis (Fig. S1A).

MFGE8 is a lactadherin preproprotein involved in recognizing and engulfing apoptotic cells, wound healing, cancer, autoimmune disease, and inflammation resolution (27). Park et al. proposed that the blood levels of MFGE8 and the lymphoid organ sources of MFGE8 ensure that the majority of cell-free HIV-1 virus in infected individuals is exposed to MFGE8, and the HIV-1 envelope phosphatidylserine (PtdSer) provides a means for MFGE8 binding, which links viral particles to the αv integrin on host cells (28). Therefore, MFGE8 possibly also mediated the rVSV-CCHFV infection in a similar way.

We then performed Western blotting to detect the endogenous expression levels of intracellular and secreted MFGE8 in these cell lines. MFGE8 showed high expression in SW-13 cells, moderate expression in A549 and A549-BAT cells, and the lowest expression level in HeLa cells (Fig. S1B). To further verify the role of MFGE8 in promoting rVSV-CCHFV infection, we overexpressed MFGE8 cDNA not only in resistant A549-BAT cells, but also in wild-type A549, HeLa, and SW-13 cells. The expression levels of MFGE8 were validated by Western blotting analysis (Fig. S1C). As expected, the empty vector control showed infection efficiency of approximately 1%–3% in A549-BAT cells, while overexpression of MFGE8 increased the viral infection to around 50% (Fig. 2E). Viral growth kinetics were analyzed by measuring the titers in the supernatants. Starting from 12 h post-infection, the supernatants of MFGE8-overexpressing A549-BAT cells exhibited significantly higher viral titers compared to vector control cells (Fig. 2F). Additionally, overexpression of MFGE8 in wild-type A549, HeLa, and SW-13 cells significantly enhanced the rVSV-CCHFV infection (Fig. 2G through I). Next, we knockout the MFGE8 in wild-type A549 cells using CRISPR/Cas9 editing system and verified the knockout efficiency by Western blotting (Fig. S1D). It showed that editing of MFGE8 significantly decreased rVSV-CCHFV infection as compared to the control (Fig. 2J). Thus, these results suggested that MFGE8 is a proviral host factor for rVSV-CCHFV infection.

### MFGE8 promotes the infection of pseudotyped, tecVLP, and authentic CCHFV across multiple strains

**Fig 3:**
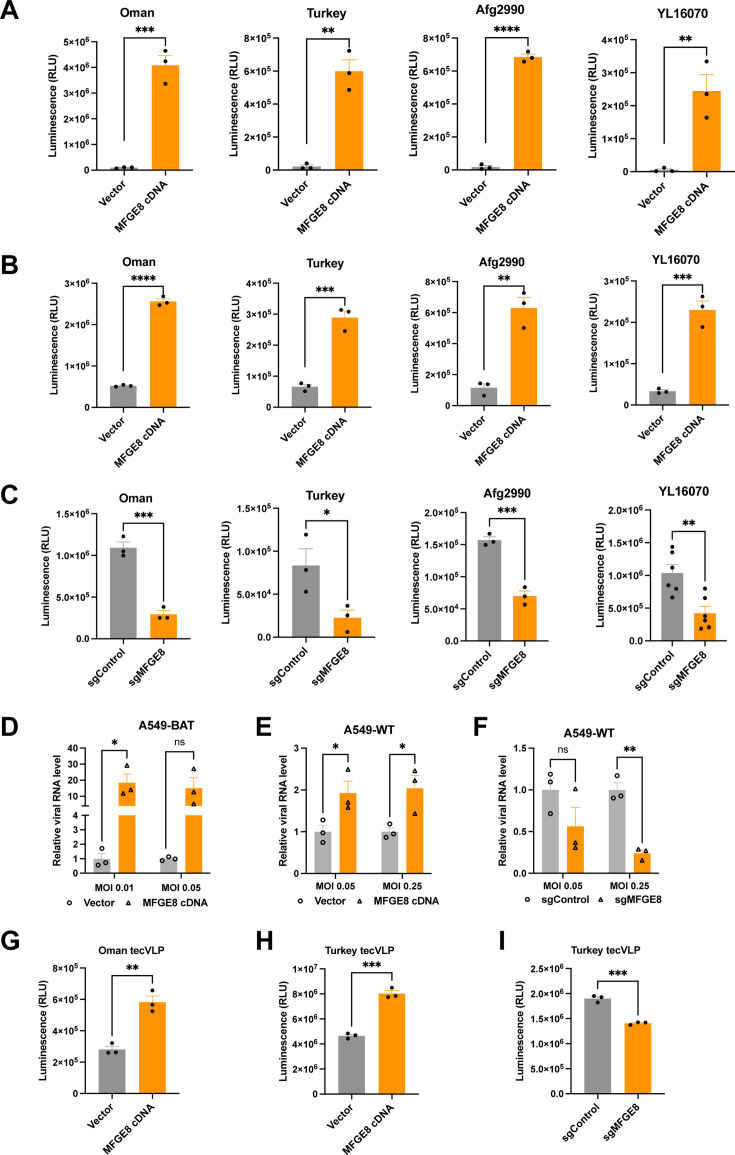
MFGE8 promotes the infection of pseudotyped, tecVLP, and authentic CCHFV across multiple strains. (**A and B**) Overexpression of MFGE8 in A549-BAT (**A**) and SW-13 (**B**) cells promotes the infection of pseudotyped CCHFV across multiple strains. The single-round VSV-based pseudovirus particles were packaged using the GPCs from different strains (Oman, Turkey, Afg2990, YL16070). The luciferase activity was determined at 24 hpi. (**C**). Knockout of MFGE8 decreases the infection of pseudotyped CCHFV across multiple strains. A549-WT cells edited with control or MFGE8-specific sgRNA were infected with pseudoviruses, and the luciferase activity was determined at 24 hpi. (**D and E**) Overexpression of MFGE8 enhances authentic CCHFV infection in A549-BAT (**D**) and A549-WT (**E**) cells. Cells were infected with CCHFV (YL16070 strain) at indicated MOIs for 72 h, and total cellular RNA were extracted for RT-qPCR. Relative viral RNA levels of S genome were analyzed by normalizing to internal *GAPDH* mRNA and then to the vector control. (**F**) Knockout of *MFGE8* decreases authentic CCHFV infection. A549-WT cells edited with control or *MFGE8*-specific sgRNA were infected with CCHFV (YL16070 strain) at indicated MOIs for 72 h. RT-qPCR analysis was performed as did for the overexpressing cells above. (**G and H**) A549-BAT cells overexpressing MFGE8 were infected with either Oman (**G**) or Turkey (**H**) tecVLPs, and NanoLuc luciferase activity was measured at 24 hpi. (**I**) *MFGE8*-knockout A549 cells were infected with Turkey tecVLP, and NanoLuc activity was measured at 24 hpi. Unpaired *t*-tests. ns, not significant; *P < 0.05; **P < 0.01; ***P < 0.001; ****P < 0.0001.

To verify whether MFGE8 promotes the infection of multiple CCHFV strains, we subsequently packaged single-round VSV-based pseudoviruses bearing the GPCs from four different strains (Oman, Turkey, Afg2990, and YL16070). As expected, overexpression of MFGE8 in A549-BAT cells significantly enhanced the luminescence readings of all four pseudovirues (Fig. 3A). Moreover, in wild-type SW-13 cells, MFGE8 overexpression could increase the infection of four CCHFV pseudoviruses (Fig. 3B). Conversely, knockout of MFGE8 in A549-WT cells obviously inhibited the infection of these pseudoviruses (Fig. 3C).

Next, we attempted to confirm the proviral function of MFGE8 using authentic YL16070 strain of CCHFV available. Through quantifying viral RNA in infected cells at 72 h post-infection, overexpression of MFGE8 in A549-BAT cells increased CCHFV infection by around 20 folds (Fig. 3D). Likewise, MFGE8 could enhance the virus infection in overexpressing A549-WT cells (Fig. 3E). Similarly, knockout of MFGE8 reduced authentic CCHFV infection (Fig. 3F). We also investigated the effects of MFGE8 on tecVLP infectivity. Overexpression of MFGE8 in A549-BAT cells significantly enhanced the infection of tecVLPs of Oman and Turkey strains (Fig. 3G and H). Moreover, knockout of MFGE8 in A549-WT cells led to reduced infection of Turkey tecVLP (Fig. 3I). Therefore, based on the results of pseudotyped, tecVLP, and authentic viruses, it suggested that MFGE8 is a common proviral host factor for CCHFV across different strains.

### MFGE8 mediates rVSV-CCHFV entry into host cells through the integrins

**Fig 4:**
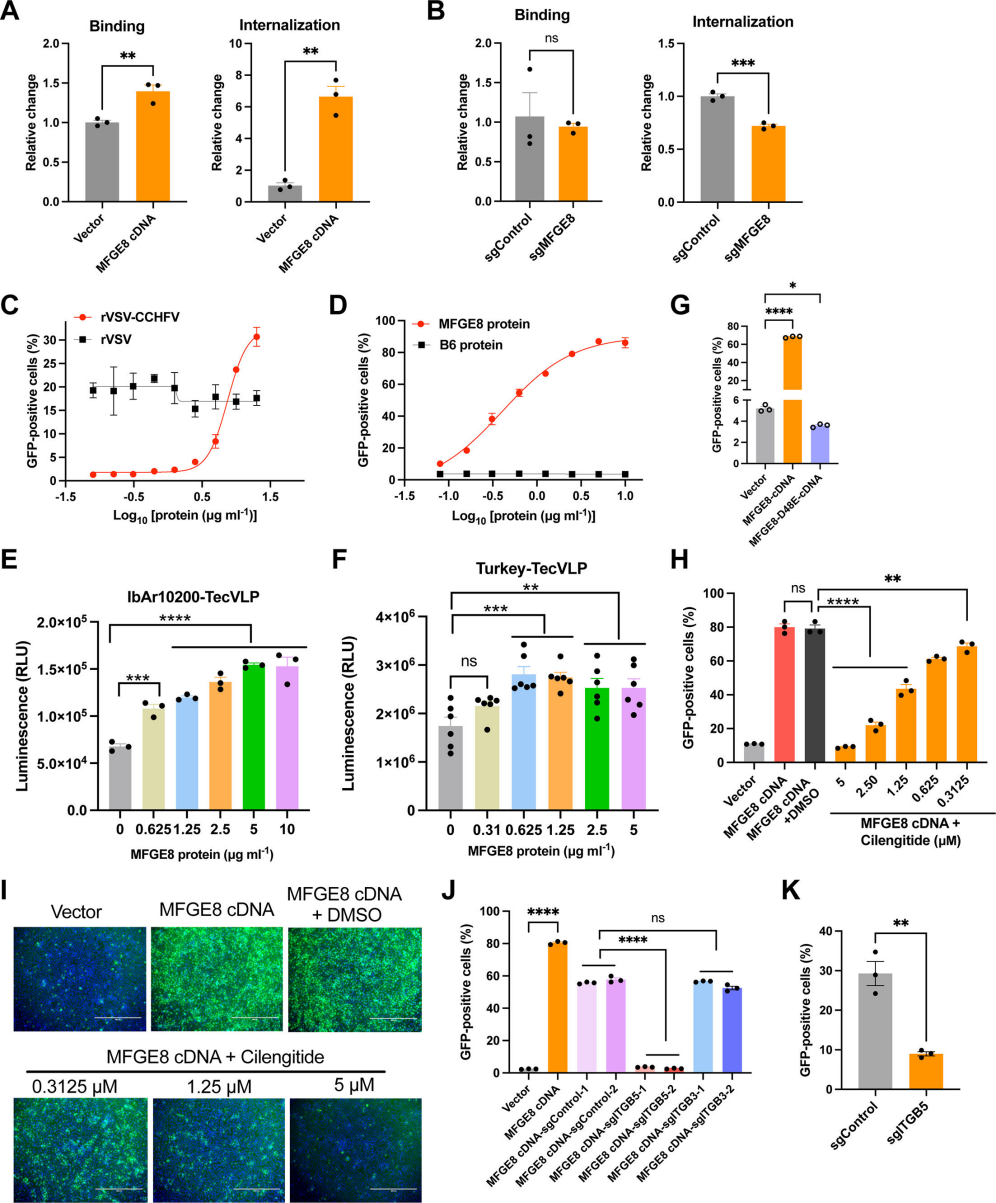
MFGE8 mediates rVSV-CCHFV entry into host cells through integrin. (**A-B**). Virus binding and internalization assays in overexpressing (**A**) or gene-edited (**B**) cells. Cells were incubated with rVSV-CCHFV (MOI 5), and the bound or internalized virions were measured by RT-qPCR for genomic RNA. The relative amount of bound or internalized virions was normalized to internal control GAPDH, and then to the control cells. (**C**). Cells pre-incubated with MFGE8 protein facilitate rVSV-CCHFV infection. A549-BAT cells were pre-incubated with different concentrations of MFGE8 protein at 37°C for 4 h, then infect with rVSV-CCHFV (MOI 3, 18 h) or rVSV (MOI 0.01, 15 h). The percentage of GFP-positive cells was analyzed by flow cytometry. (**D**). rVSV-CCHFV particles pre-incubated with MFGE8 protein facilitate virus infection. The rVSV-CCHFV particles (MOI 3) were pre-incubated with different concentrations of MFGE8 protein or mpox virus B6 control protein at 37°C for 1 h, then inoculated the A549-BAT cells for 18 h. The percentage of GFP-positive cells were analyzed by flow cytometry. (**E and F**) TecVLPs pre-incubated with MFGE8 protein facilitates virus infection. TecVLPs of IbAr10200 or Turkey stain were pre-incubated with varying concentrations of MFGE8 protein at 37°C for 1 h, then used to infect A549-BAT cells pre-transfected with plasmids expressing the L and N proteins. After 15 h of infection, NanoLuc activity was quantified. (**G**). Overexpression of MFGE8 with D48E mutation decreases the rVSV-CCHFV (MOI 3, 20 h) infection in A549-BAT cells. The percentage of GFP-positive cells were analyzed by flow cytometry. (**H-I**). The inhibitor of integrins decreases the rVSV-CCHFV infection. The MFGE8-overexpressing A549-BAT cells were pre-treated with integrin inhibitor cilengitide for 2 h at 37°C, and then infected with rVSV-CCHFV (MOI 3, 20 h) for flow cytometry analysis (**H**). The representative images were taken with fluorescence microscope (**I**). Scale bar, 400 µm. (**J**). MFGE8-overexpressing A549-BAT cells were edited with control, ITGB5-, or ITGB3-specific sgRNAs, and infected with rVSV-CCHFV (MOI 3, 17 h), followed by flow cytometry analysis. (**K**). A549-WT cells edited with control or ITGB5 sgRNA, followed by infection with rVSV-CCHFV (MOI 0.5, 16 h) for flow cytometry analysis. Unpaired *t*-tests (**A, B, K**), One-way ANOVA with Dunnett’s multiple comparisons test (**E, F, G, H, J**). ns, not significant; *, *P*  <  0.1, **, *P*  <  0.01; ***, *P*  <  0.001; ****, *P*  <  0.0001.

Given the enhancement of MFGE8 for rVSV-CCHFV but not rVSV infection as described above in Fig. 2B and C, we speculated that MFGE8 affects the cell entry stage of rVSV-CCHFV that bears the GPC. The binding and internalization assays were performed, and indicated that more rVSV-CCHFV virions bind and enter MFGE8-overexpressing A549-BAT cells. Compared with vector control cells, overexpression of MFGE8 promoted the binding by 1.5 times and internalization by seven times (Fig. 4A). In MFGE8-editied A549-WT cells, the internalized rVSV-CCHFV was decreased by approximately 1.3 times as compared to the sgRNA control cells (Fig. 4B). The binding of virions in MFGE8-edited cells was not affected, probably due to the strong non-specific binding in A549-WT cells that express an abundance of attachment factors, such as heparan sulfate.

It has been shown that pre-incubation with recombinant MFGE8 protein can promote the transduction of lentiviruses packaged with alphavirus or baculovirus glycoproteins, via bridging the binding to PtdSer on the lentivirus envelope and to the integrins on cell surface (29). We investigated whether the recombinant MFGE8 protein can also increase the infection of rVSV-CCHFV. We pre-incubated A549-BAT cells with MFGE8 protein, starting at a concentration of 20 µg/mL with a serial of twofold dilutions, followed by infection with rVSV-CCHFV. It showed that the highest concentration of MFGE8 protein could increase the infection efficiency by about 30 times for rVSV-CCHFV but has no effect on rVSV infection (Fig. 4C). Similarly, we found that pre-incubation of MFGE8 protein with rVSV-CCHFV can markedly improve the infection efficiency (Fig. 4D). As negative control, the mpox virus B6 protein had no effect on rVSV-CCHFV infection (Fig. 4D). We also found that MFGE8 protein can promote the infection of IbAr10200 and Turkey tecVLPs in a dose-dependent manner (Fig. 4E and F).

MFGE8 has been reported to bind to integrin αVβ3 or αVβ5 receptor (27). Integrins recognize Arg-Gly-Asp (RGD) motif in their physiological ligands (30), and the epidermal growth factor (EGF) domain of MFGE8 displays an RGD motif (31, 32). We attempted to explore whether MFGE8 promotes rVSV-CCHFV infection through integrins. Interestingly, in contrast to the wild-type MFGE8 that enhances the infection as presented above, overexpression of D48E mutant that disrupts the “RGD” motif in the EGF domain of MFGE8 resulted in a significant reduction of rVSV-CCHFV infection (Fig. 4G). The expression of MFGE8 mutant was validated by Western blotting analysis (Fig. S1E). We also used the Cilengitide, a cyclized RGD-containing pentapeptide that potently and selectively inhibits αvβ3 and αvβ5 integrins, to examine its effect on MFGE8-mediated infection. It showed that around 0.3 µM of Cilengitide could efficiently dampen the increased infection by MFGE8 overexpression, while 5 µM completely inhibited the proviral function of MFGE8 (Fig. 4H and I). The cytotoxicity of Cilengitide on cells was checked (Fig. S2).

To further investigate which integrin mediates the proviral function of MFGE8, we knocked out *ITGB5* and *ITGB3* in MFGE8-overexpressing A549-BAT cells, respectively. The results showed that *ITGB5* knockout completely abolished the proviral effect of MFGE8, whereas *ITGB3* knockout had subtle impact on infection efficiency compared to the sgControl group (Fig. 4J). This indicated that MFGE8 promotes CCHFV entry into host cells primarily through ITGB5, not ITGB3. Moreover, editing of ITGB5 with specific sgRNA significantly inhibited the rVSV-CCHFV infection in A549-WT cells (Fig. 4K). The above experiments indicated that the promotion of viral infection by MFGE8 is mediated by integrins.

### MFGE8 binds directly to the Gc protein of CCHFV

**Fig 5:**
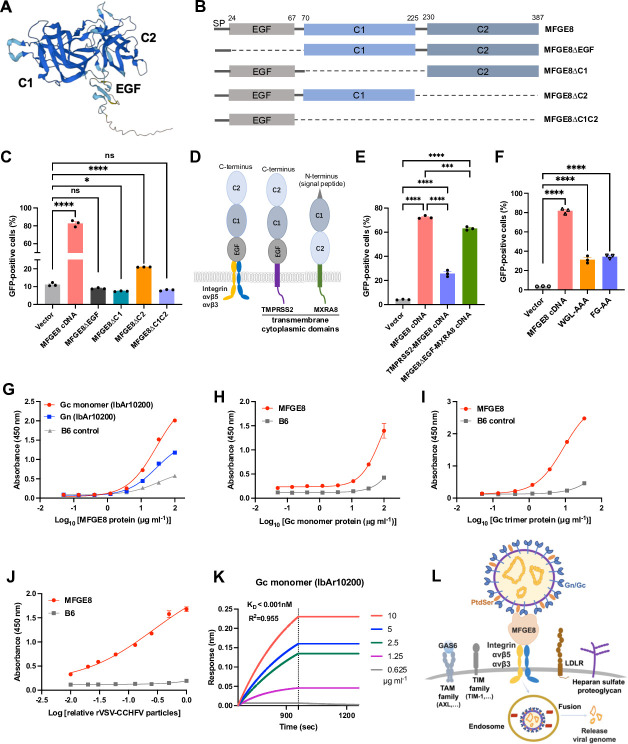
MFGE8 directly binds to the Gc domain of CCHFV. (**A**) The structure of MFGE8 protein predicted by AlphaFold2. Three domains, EGF, C1, C2, are indicated. (**B**) Schematic of different truncations of MFGE8 protein. (**C**) Overexpression of different truncated forms of MFGE8 in A549-BAT cells, followed by infection with rVSV-CCHFV (MOI 3, 20 h) and flow cytometry analysis. (**D**) Schematic of chimeric MFGE8 proteins. The full-length MFGE8 without signal peptide was fused with the type II transmembrane protein, TMPRSS2. The C1 and C2 domains of MFGE8 were fused with type I transmembrane protein, MXRA8. The extracellular region of TMPRSS13 or MXRA8 was replaced. (**E**). Overexpression of chimeric MFGE8 proteins in A549-BAT cells, followed by infection with rVSV-CCHFV (MOI 3, 20 h) and flow cytometry analysis. (**F**) Overexpression of different mutants of MFGE8 in A549-BAT cells, followed by infection with rVSV-CCHFV (MOI 3, 20 h) and flow cytometry analysis. One-way ANOVA with Dunnett’s multiple comparisons test (C, E, and F). ns, not significant; *P < 0.1; ***P < 0.001; ****P < 0.0001. (**G**) Binding of MFGE8 protein to CCHFV Gc monomer, Gn, and Mpox virus B6 control protein. These proteins were pre-coated onto ELISA plates at a concentration of 10 µg/mL, followed by incubation of different concentrations of MFGE8 protein. The binding was evaluated using anti-MFGE8 and HRP-conjugated secondary antibodies. (**H–K**) MFGE8 and B6 control proteins were coated onto ELISA plates at a concentration of 10 µg/mL. Then, different concentrations of CCHFV Gc monomer (**H**), Gc trimer (**I**), or rVSV-CCHFV particles (**J**) were incubated and detected using anti-CCHFV-Gc ADI-36121 antibody and HRP-conjugated secondary antibody. (**K**) The binding affinity of MFGE8 to CCHFV Gc domain. His-tagged MFGE8 protein was immobilized onto the Ni-NTA biosensors. Binding parameters of recombinant Gc monomer to MFGE8 protein were measured by biolayer interferometry (BLI). Using a 1/1 binding model to derive equilibrium dissociation constant (Kd) values. (**L**) The model of soluble MFGE8-mediated cell entry of CCHFV. The soluble MFGE8 protein binds directly to both Gc protein and phosphatidylserine (PtdSer) on the surface of CCHFV virions and acts as a bridge by binding to the integrins on the cell membrane to promote virus infection.

MFGE8 has a signal peptide located at the N-terminus, which plays a crucial role in guiding it into the secretion pathway. MFGE8 also possesses an EGF domain, followed by two coagulation domains (C1 and C2) (Fig. 5A). To identify critical regions of MFGE8 that are required for rVSV-CCHFV infection, we generated truncated variants lacking ΔEGF, ΔC1, ΔC2, or ΔC1C2 and validated the expression of ΔEGF and ΔC2 by Western blotting analysis but not ΔC1 and ΔC1C2 because the antibody only recognizes the C1 domain (Fig. 5B; Fig. S1F). Overexpression of truncated variants in A549-BAT cells showed that, compared to full-length MFGE8, ΔEGF completely lose their ability to promote rVSV-CCHFV infection. ΔC1 and ΔC1-C2 possibly also abolished the function, given the uncertainty of their expression. While ΔC2 showed less pronounced proviral effects compared to full-length MFGE8, it still increased infectivity two-fold (Fig. 5C). The deletion of EGF domain disrupts the interaction between MFGE8 and integrins, while the deletion of C1 and/or C2 domains may impair the binding to the virus. These results indicated that the three domains of MFGE8 are all possibly required for promoting rVSV-CCHFV infection.

To further assess the function of soluble MFGE8, we engineered chimeric MFGE8 proteins to render their expression on the plasma membrane (Fig. 5D). The soluble MFGE8 without the signal peptide was fused with a type II transmembrane protein, TMPRSS2, to replace the extracellular region. Similarly, the C1 and C2 domains of MFGE8 were fused with a type I transmembrane protein, MXRA8, to replace the extracellular region. TMPRSS2 and MXRA8 are two cell surface proteins required for coronavirus and alphavirus entry, respectively (33, 34). The two engineered MFGE8 proteins were expressed and displayed on the cell surface similar to the soluble MFGE8 that is anchored to the surface through the integrins. Expression of engineered MFGE8 proteins in A549-BAT cells significantly enhanced rVSV-CCHFV infection, particularly the display of C1 and C2 domains on the cell surface in the backbone of MXRA8 (Fig. 5D).

It has been shown that MFGE8 is a secreted protein that binds to the PtdSer through its C1 and C2 domains (32). Shao et al. further confirmed that, by mutating the “WGL” at amino acids 26-28 of MFGE8 to “AAA” or the “FG” at amino acids 81-82 to “AA,” the binding affinity between MFGE8 and membrane PtdSer decreased over 90% (35). To determine whether MFGE8 promotes rVSV-CCHFV infection by binding to the PtdSer on the evelople of viral particles, we constructed “WGL-AAA” and “FG-AA” mutants of MFGE8 for overexpression and validated by Western blot analysis (Fig. S1G). When compared to the full-length MFGE8, the mutants decreased the virus infection from 80% to 30% (Fig. 5F), suggesting that the binding of MFGE8 to PtdSer plays a significant role. However, the infection efficiency for mutants was still around 10-fold higher than the vector control (Fig. 5F).

The unexpected high infection efficiency for MFGE8 mutants that disrupt the binding to PtsSer implied that MFGE8 may also bind to other components of virions, such as the Gc protein that is exposed on the virion surface for receptor binding. To test this hypothesis, we expressed and purified Gc and Gn proteins of CCHFV (Fig. S3) and coated for ELISA-based binding assay. Interestingly, we found that MFGE8 is captured by the Gc monomer protein, with much higher binding ability than the Gn protein (Fig. 5G). An unrelated viral protein, mpox virus surface B6 protein, was used as control. Additionally, the MFGE8 or B6 control protein was coated as bait, and the increasing concentrations of Gc monomer or trimer protein was added for capture. The Gc trimer assembles in the endosome to facilitate membrane fusion. As expected, both Gc monomer and trimer could apparently bind to the MFGE8, but not B6 protein (Fig. 5H and I). Likewise, the rVSV-CCHFV particles could bind to the MFGE8 protein (Fig. 5J). What’s more, biological layer interferometry showed that MFGE8 protein can efficiently bind to the Gc monomer of CCHFV, with the *K*_d_ < 0.001 nM (Fig. 5K). *In silico* analysis revealed that both C1 and C2 regions of MFGE8 contribute to binding to CCHFV Gc trimer (Fig. S4).

In summary, we demonstrated that the enhancement of CCHFV infection by secretory MFGE8 protein may be the synergistic effect of binding to both PtdSer and Gc protein on the surface of CCHFV virions (Fig. 5L). The soluble MFGE8 acted as a bridge by binding to the virions and to the integrins on the cell plasma membrane to mediate the cell entry of CCHFV (Fig. 5L).

## DISCUSSION

CCHFV infects various animals, and humans suffer from serious diseases. Elucidating the cell entry process of CCHFV and identifying host factors that are required for entry will be of significance to understand the tissue tropism and pathogenesis and to develop antivirals targeting the invasion. Here, we performed genome-wide CRISPR knockout screen and identified some common host factors that are important for CCHFV entry. Additionally, we performed genome-wide CRISPR activation screen and identified the MFGE8 as a proviral host factor for CCHFV. Overexpression of MFGE8 could significantly promote the infection of pseudotyped, tecVLP, and authentic CCHFV, while knockout of MFGE8 decreased the infection. Pre-treatment of cells with recombinant MFGE8 protein or pre-incubation with virus could dose-dependently increase the rVSV-CCHFV and tecVLP infection. More importantly, we demonstrated that, in addition to the binding ability with PtdSer as reported previously, MFGE8 also can bind directly to the CCHFV Gc protein. The binding to both molecules on the virion surface will apparently enhance the cell entry into cells.

Studies have shown that integrins play important roles in mediating the entry of a wide range of viruses, mostly via the direct interaction with a RGD motif exposed on the surface proteins of virions, such as SARS-CoV-2 (30), Zika (36), Foot and mouth disease virus (FMDV) (37), Varicella Zoster Virus (38), herpes simplex virus (HSV) (39), Hantaan (40), and other viruses (41). However, the integrins can also bind to host proteins, such as the soluble MFGE8 that possesses the RGD motif in the EGF domain, serving as a bridge between viruses and cells for entry. It has been shown that MFGE8 can enhance the transduction with lentiviruses pseudotyped with various envelope proteins, including Sindbis virus, Ross River virus, and baculovirus (gp64), through the binding to PtdSer on virus envelope, and to the integrins on the cell surface (29). As for CCHFV studied here, MFGE8 functions as an entry factor through similar mechanism of action. The exceptional finding in MFGE8 can also bind to the CCHFV Gc protein. It is noteworthy that both ITGAV and ITGB5 which form as the integrin αVβ5 heterodimer were identified from our initial CRISPR knockout screen, and proviral function of ITGB5 was validated. Whether the integrins can also directly interact with surface Gc protein to mediate the CCHFV entry needs to be further studied.

LDLR was recently identified as a receptor for CCHFV (14–16). Pre-treatment with anti-LDLR monoclonal antibodies can dose-dependently reduce CCHFV infection in primary HUVECs, with limited inhibitory effect on primary human PBMCs (14), suggesting that there may be other receptors or co-factors present in specific types of cells for CCHFV infection. It also revealed that the exchangeable host ApoE incorporated into CCHFV particles mediates the interaction of virions with receptor LDLR or LPR8, to facilitate CCHFV entry into cells (15, 16). In this study, the identification of soluble MFGE8 represents another mode of CCHFV entry, which is very similar to the Gas6 protein via the TAM receptors (Tyro3, Axl, and Mer) employed by many enveloped viruses (21, 29, 42–44).

CCHFV replicates efficiently in cells such as epithelial cells, dendritic cells, and tissue resident macrophages. The productive infection of these cells promotes the spread of the virus and leads to early infection of local lymph nodes and peripheral blood monocytes, supporting the systemic transmission of the virus (45–47). MFGE8 is widely expressed in the liver, kidney, intestine, lung, mammary gland, brain, heart, spleen, and reproductive organs. After CCHFV infection, the liver is one of the main target organs. The highly expressed MFGE8 in the liver may directly interact with the CCHFV surface glycoprotein Gc and PtdSer, assisting in the internalization of the virus. Additionally, due to the wide distribution of cell types expressing the integrins, the viral particles captured by soluble MFGE8 in blood may significantly expand the tissue tropism of CCHFV, leading to the occurrence of systemic infection. Moreover, MFGE8 is a component of the milk (48). Although some studies indicated that breastfeeding transmission or virus in milk was not detected (49, 50), whether MFGE8 plays a role in promoting the transmission via breast milk warrants further study.

The wide geographical distribution and large population of CCHFV infection urge the need to explore the host determinants of the tropism and pathogenesis. More rapid and reliable diagnosis, as well as effective vaccines and antiviral drugs, are needed to limit the burden of CCHFV on patients and public health. Our study identified a soluble host factor MFGE8 involved in the cell entry of CCHFV, and the strategies developed to disturb the interaction of MFGE8 with virions or with integrins may help to prevent and control the endemic of CCHFV in high-risk areas. Our study also provides new insight into the underlying mechanisms of cell entry of CCHFV.

## MATERIALS AND METHODS

### Cells and viruses

Vero E6 (Cell Bank of the Chinese Academy of Sciences, Shanghai, China), A549 (ATCC #CCL-185), HeLa (ATCC #CCL-2), SW13 (TCHu221), HEK 293T (ATCC # CRL-3216) all were cultured at 37°C in Dulbecco’s modified Eagle’s medium (Hyclone #SH30243.01) supplemented with 10% fetal bovine serum (FBS), 10  mM HEPES, 1  mM sodium pyruvate, 1 × non-essential amino acids, and 100  U/mL of penicillin–streptomycin. A549-BAT is a clonal cell line generated in our laboratory that B3GAT3, AXL, and TIM1 are deficient. All cell lines were routinely screened for mycoplasma contamination and found to be negative. The recombinant vesicular stomatitis virus expressing the GFP protein (rVSV-GFP, thereafter rVSV) and replication-competent VSV-based CCHFV pseudovirus (rVSV-CCHFV) carrying both CCHFV glycoprotein precursor (GPC) and GFP reporter were generated previously (17) and propagated and titrated in BHK-21 cells. CCHFV authentic virus (YL16070 strain), was propagated in Vero E6 cells and titrated in SW13 cells. All experiments involving CCHFV authentic virus were performed in the biosafety level 3 (BSL-3) facility of Wuhan Institute of Virology, Chinese Academy of Sciences, following the regulations.

### CRISPR screens

The human Brunello CRISPR knockout pooled library targeting 19,114 genes (Addgene #73178) or Calabrese activation pooled library targeting 18,885 genes (Addgene #92379) was a gift from David Root and John Doench (51) and packaged in 293 FT cells after co-transfection with psPAX2 (Addgene #12260) and pMD2.G (Addgene #12259) using FugeneHD (Promega). At 48 h post transfection, supernatants were harvested, clarified by spinning at 3,000 rpm for 15 min, filtered, and aliquoted for storage at −80°C.

A549-Cas9 cells were generated by transduction of wild-type A549 with a packaged lentivirus lentiCas9-Blast (Addgene #52962). A549-BAT-dCas9 cells were generated by transduction of A549-BAT with a packaged lentivirus lenti dCAS-VP64_Blast (Addgene #61425). A549-Cas9 or A549-BAT-dCas9 cells were transduced with respective knockout or activation library at a multiplicity of infection (MOI) of ~0.3 by spinoculation at 1000 g and 32°C for 30 min in 12-well plates, followed by selection with puromycin for around 7 days. For knockout screen, cells were inoculated with rVSV-CCHFV pseudovirus (MOI 3) and incubated until nearly all cells were killed. The medium was changed, and remaining live cells grew to form colonies. The cells were then harvested and re-plated to the flasks. For activation screen, cells were inoculated with rVSV-CCHFV pseudovirus (MOI 3) for 24 h, and cells were harvested and sorted for the GFP positive population. Genomic DNA from surviving or sorted cells and uninfected cells was extracted for sgRNA amplification and next generation sequencing using an Illumina NovaSeq 6000 platform. The sgRNA sequences targeting specific genes were trimmed using the FASTX-Toolkit (http://hannonlab.cshl.edu/fastx_toolkit/) and cutadapt 1.8.1 and further analyzed for sgRNA abundance and gene ranking by a published computational tool (MAGeCK) (see Table S1).

### Generation of overexpressing and knockout cells

To construct the overexpressing cells, human MFGE8 cDNA was purchased from SinoBiological (NM_005928.1) and cloned into pLV-EF1α-IRES-Puro vector (Addgene #85132). The full-length MFGE8 without signal peptide sequences were amplified and fused with TMPRSS2 gene to replace the extracellular region. Similarly, the C1 and C2 domains of MFGE8 were amplified and fused with MXRA8 gene to replace the extracellular region. The resulting lentivectors were co-transfected with helper plasmids psPAX2 (Addgene #12260) and pMD2.G (Addgene #12259) at a ratio of 2:2:1 into 293T cells to package the lentivirus. After 48 h, the cell supernatant was collected. Transduction of lentivirus into A549-BAT, A549, Hela, and SW13 cells was performed to overexpress the MFGE8 protein. After screening with puromycin for 7 days, the surviving cells were used for Western blotting validation of overexpression. To construct the knockout A549 cells, sgRNAs targeting the human *MFGE8*, *ITGB3*, and *ITGB5* were synthesized and cloned into lentiCRISPR v2 (Addgene #52961) vector. The packaging of lentivirus, transduction, and puromycin selection is the same as the construction of overexpression cells above. The sgRNA sequences were listed in Table S2.

### Virus binding and internalization assay

For binding assay, A549-BAT or A549-WT cells were seeded in a 24-well plate. The next day, the plate with confluent cells was placed on ice for 15 min, washed twice with pre-cooled PBS, and then incubated with rVSV-CCHFV virus (MOI 5). After being placed on ice for 45 min, cells were washed five times with pre-cooled PBS and then lysed with RL lysis buffer (TIANGEN # DP430) for RNA extraction. For the internalization assay, after virus binding and wash as described above, cells were added with 2% FBS DMEM and placed in a 37°C incubator for 45 min. Uninternalized virions on the cell surface were removed by treating cells with 400 µg/mL protease K on ice for 45 min. After three washes, cells were lysed by RL lysis buffer for RNA extraction. The One Step PrimeScript RT-PCR Kit (Takara #RR064A) was used for RT-qPCR. The relative amount of bound or internalized virions was normalized to internal control GAPDH, followed by normalizing to the control cells. The primer sequences were listed in Table S2.

### Flow cytometry

The rVSV or rVSV-CCHFV infected cells were washed once with PBS, trypsinized, and then inactivated with 10% FBS DMEM. The cells were fixed with 2% PFA for 10 min and then centrifuged at 1,500 rpm for 5 min. The supernatant was discarded, and the cells were resuspended, washed with PBS, and subjected to flow cytometry (Thermo, Attune NxT). The percentage of GFP-positive cells was analyzed using the FlowJo v10.0.7.

### Packaging of single-round VSV pseudotyped with the GPCs from different CCHFV strains

The optimized GPC sequences of Oman (KR864901), Turkey (KR864902), Afg2990 (HM452306.1), and YL16070 (KY354082) were cloned into pCAGGS vector. 293T cells were transfected with plasmids for 24 h. After washing, cells were infected for 2 h with single-round VSV^ΔG^-Nluc-GFP virus (17) and then washed three times, followed by incubation with 2% FBS DMEM containing anti-VSV-G neutralizing antibody. After 24 h, the supernatants were collected and stored at −80°C for use.

### Packaging of transcription- and entry-competent virus-like particles of CCHFV

BHK-T7 cells were seeded in 6-well plates and cultured overnight. Cells were co-transfected with the minigenome plasmid and helper plasmids (pCAGGS-CCHFV-N, pCAGGS-CCHFV-L, pCAGGS-CCHFV-GPC) using Fugene HD transfection reagent. At approximately 16–18 h post-transfection, the transfection medium was discarded and replaced with fresh complete medium. Three days post-transfection, tecVLP-containing supernatants were harvested and stored at −80°C.

### Plaque-forming assay

Briefly, Vero cell monolayers in 96-well plates were inoculated with serially diluted virus for 2 h and then overlaid with methylcellulose for 48 h. Cells were fixed with 2% PFA for 1 h, stained with crystal violet at room temperature for 15 min, and plaques were counted under microscopy.

### Protein blocking assay

Human recombinant MFGE8 protein with His tag at the C-terminus was purchased from SinoBiological (10853-H08B). The MFGE8 protein concentration was twofold diluted starting from 20 µg/mL. A549-BAT cells were incubated with different concentrations of MFGE8 protein at 37°C for 4 h and then washed once with PBS, followed by infection with rVSV-CCHFV virus (MOI 3). Cells were collected for flow cytometry analysis after 20 h. Alternatively, different concentrations of MFGE8 protein were pre-incubated with rVSV-CCHFV virus at 37°C for 1 h and then added to A549-BAT cells for infection for 20 h. Cells were then collected for flow cytometry analysis. For assays with tecVLP infection, A549-BAT cells in 6-well plates were transfected with pCAGGS-L and pCAGGS-N for 20 h. Cells were trypsinized and seeded into 96-well plates. TecVLPs were pre-incubated with serial dilutions of recombinant MFGE8 protein (0–10 μg/mL) for 1 h at 37°C prior to infection. At 15 h post-infection, cells were lysed with Nano-Glo Luciferase Lysis Buffer (Promega, Cat# N1120) for 10 min at room temperature. Luminescence was quantified using the FlexStation 3 (Molecular Devices).

### Cell viability assay

Cells were seeded in 96 well plates with a density of 5 × 10^4^. The next day, cells were treated with Cilengitide at the concentrations of 0, 0.625, 1.25, 2.5, 5, and 10 µM. After 24 h, 100 µL of the CellTiter-Lumi Luminescence-based Cell Vitality Detection Kit (Beyotime #C0065M) was added to each well. After incubation for 10 min at room temperature, luminescence was recorded by using a FlexStation 3 (Molecular Devices) with an integration time of 1 s per well.

### Integrin inhibition assay

A549-BAT were seeded in 96 well plates with a density of 5 × 10^4^. The next day, cells were treated with different concentrations of Cilengitide to inhibit the αvβ3 and αvβ5 for 2 h at 37°C, and DMSO was used as control. Cells were then infected with rVSV-CCHFV virus (MOI 3) in the presence of inhibitor. After 20 h, cells were collected for flow cytometry analysis.

### Authentic CCHFV infection

A549-BAT or A549-WT cells in 12-well plates were inoculated with authentic CCHFV (YL16070 strain, GenBank accession number: KY354082) at MOI of 0.01–0.25, and cells were lysed at 72 h post-infection. The virus replication was determined by analyzing the copies of S genome using RT-qPCR. The primer sequences were listed in Table S2.

### Western blotting

Cells were lysed in RIPA buffer (Beyotime #P0013B) containing protease inhibitors (Sigma-Aldrich #S8830). Cell lysates were clarified by centrifugation at 12,000 rpm for 10 min at 4°C. Samples were denatured at 95°C for 10 min, in reducing loading buffer (50 mM Tris, pH 6.8, 10% glycerol, 2% SDS, 0.02% [wt/vol] bromophenol blue, 100 mM DTT) and electrophoresed in 10% SDS polyacrylamide gels, and proteins were transferred to PVDF membranes. Membranes were blocked with 5% non-fat dry powdered milk in TBST (100 mM NaCl, 10 mM Tris, pH7.6, 0.1% Tween 20) for 1 h at room temperature and then incubated with primary antibodies to detect MFGE8 (MFG-E8 monoclonal antibody, Proteintech, #67797-1-PBS, 1:3000) or actin (Beta Actin polyclonal antibody, Proteintech, # 20536-1-AP, 1:3,000) at 4°C overnight. After three washes with TBST, the membrane was incubated with horseradish peroxidase (HRP)-conjugated secondary antibodies at room temperature for 1 h. The membrane was washed again with TBST three times, each time for 10 min, and developed using SuperSignal West Pico or Femto chemiluminescent substrate according to the manufacturer’s instructions (Thermo Fisher).

### Protein expression and purification

Expression plasmids based on pCAGGS vector were constructed by inserting CCHFV Gc10200 aa 1,041–1,579, Gn10200 aa 520–690, and mpox virus B6 aa 1–279 fused with the Strep-Tag II at the C-terminus. On the day before the experiment, FreeStyle 293 F cells were seeded at a density of 5 × 10^5^. The next day, expression plasmids were transfected using EZ- *Trans* transfection reagent (AC04L092). The supernatants were collected 4 days after transfection, centrifuged at 8,000 × g for 30 min, and used the Strep-TactinXT 4Flow resin for purification. The purified protein was dialyzed in PBS, filtered through a 0.2 µm filter, and stored at −80°C. Protein purity was confirmed through SDS-PAGE and Coomassie Brilliant Blue staining.

### ELISA-based protein-binding assay

96-well EIA/RIA high protein-binding affinity plate (Corning) was coated with purified CCHFV Gn, Gc monomer, or B6 protein (10 µg/mL per well) overnight at 4°C and blocked with 4% bovine serum albumin in PBS. Blocking buffer was removed by washing five times with PBST. MFGE8 protein was added at different concentrations and incubated at room temperature for 1 h. After five washes with PBST, primary antibody (MFG-E8 Monoclonal antibody, Proteintech #67797-1-PBS, 1:3,000) was incubated at room temperature for 1 h. After another five washes with PBST, HRP-conjugated secondary antibody was incubated at room temperature for 1 h. The 3,3′,5,5′-Tetramethylbenzidine (TMB) substrate (Invitrogen #34028) was added to each well at a volume of 100  µL and left for 15 min at room temperature to allow the colorimetric reaction to occur. The reaction was stopped by addition of 100  µL of 2 M H_2_SO_4_. Absorbances were read by an automated plate spectrometer at a wavelength of 450 nm and analyzed using SoftMax Pro software. Conversely, the plates were coated with MFGE8 or B6 control protein (10 µg/mL per well) overnight at 4°C and bound with different concentrations of Gc monomer, Gc trimer protein, or rVSV-CCHFV particles. Similar procedure was performed as described above.

### Bio-layer interferometry

The binding affinity between MFGE8 and Gc monomer of the CCHFV IbAr10200 strain was measured using the ForteBio Octet Red system (ForteBio, Inc). The His-tagged MFGE8 protein (15 µg/mL) was immobilized onto the NTA biosensors; the association and dissociation of the indicated concentrations of Gc protein to MFGE8 were monitored in 200  µL of PBS containing 0.02% Tween 20 and 0.1%BSA. The binding curves and kinetics of association and dissociation were analyzed using the ForteBio Data Analysis Software.

### Statistical analysis

GraphPad Prism version 10.3.1 software was used for graphical representation and statistical analysis. Two-tailed unpaired *t*-tests or two-way analysis of variance (ANOVA) with Sidak’s multiple-comparison test or Dunnett’s multiple-comparison test were used for data analysis. *P* values under 0.05 were considered statistically significant and the following denotations were used: ****P < 0.0001; ***P < 0.001; **P < 0.01; *P < 0.05; ns (not significant), *P* > 0.05.

## Data Availability

The authors declare that all relevant data supporting the findings of this study are available within the paper and its Supplementary information. The Supplemental Data provide information for the CRISPR screens, qRT-PCR, and sgRNA sequences.

## SUPPLEMENTAL MATERIAL

Supplemental material mbio.01617-25-s0001.pdf Figures S1 to S4 and captions for supplemental tables.

Table S1 mbio.01617-25-s0002.xlsx List of genes and scores after MaGeck analysis of CRISPR screens.

Table S2 mbio.01617-25-s0003.xlsx List of primers and probes used for RT-qPCR experiments, and sgRNA sequences of genes selected for validation experiments.

ASM does not own the copyrights to Supplemental Material that may be linked to, or accessed through, an article. The authors have granted ASM a non-exclusive, world-wide license to publish the Supplemental Material files. Please contact the corresponding author directly for reuse.
