# Supplementary material for: Soluble MFGE8 mediates cell entry of Crimean-Congo hemorrhagic fever virus
Source: mBio. 2025 Aug 25;16(10):e01617-25. doi: 10.1128/mbio.01617-25 (PMC12505966; doi:10.1128/mbio.01617-25)
Supplement: Supplemental material — Figures S1 to S4 and captions for supplemental tables. [file mbio.01617-25-s0001.pdf]

SUPPLEMENTARY FIGURES LEGENDS

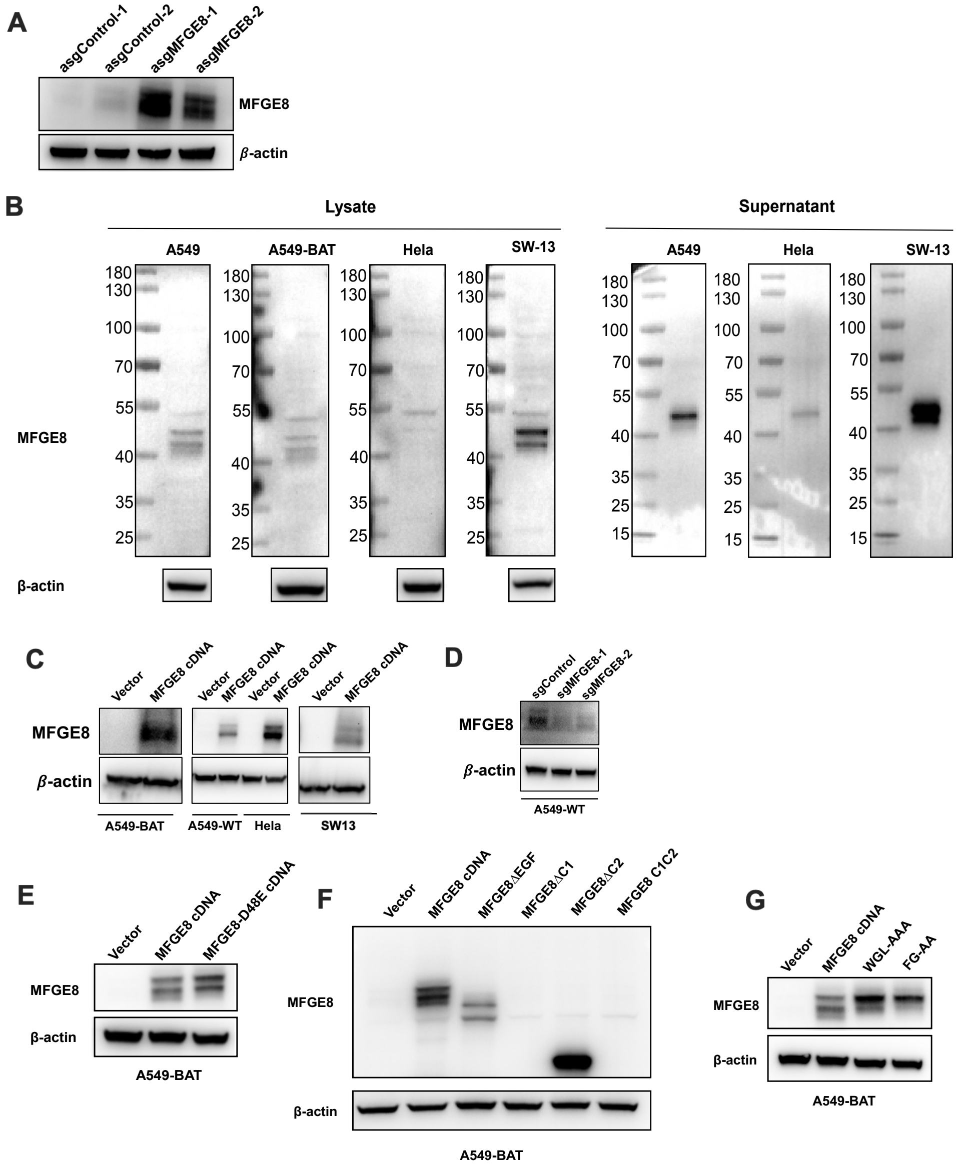

Supplementary Figure 1. Western blotting of MFGE8 proteins. A. Expression of endogenous MFGE8 was activated using two control or MFGE8-specific sgRNAs in A549-BAT cells. Protein expression levels were probed with anti-MFGE8 or internal control anti-actin antibodies. **B**. The endogenous expression of intracellular and secreted MFGE8 in various cell lines were assessed by Western blotting analysis using an anti-MFGE8 antibody. **C**. MFGE8 cDNA was overexpressed in A549-BAT, A549-WT, HeLa, or SW-13 cells, and expression levels were evaluated with anti-MFGE8 or internal control anti-actin antibody. **D**. A549-WT cells were edited with control or two

MFGE8-specific sgRNAs, and expression levels were examined with anti-MFGE8 or internal control anti-actin antibodies. **E**. MFGE8 and its D48E mutant cDNA were overexpressed in A549-BAT cells, and expression levels were evaluated with anti-MFGE8 or internal control anti-actin antibody. **F**. MFGE8 or its different truncations were overexpressed in A549-BAT cells, and expression levels were examined with anti-MFGE8 or internal control anti-actin antibody. **G**. MFGE8, MFGE8-WGL-AAA, and MFGE8-FG-AA mutants were overexpressed in A549-BAT cells, and expression levels were evaluated with anti-MFGE8 or internal control anti-actin antibody.

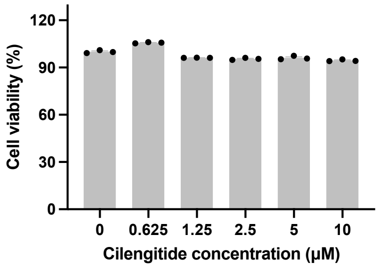

Supplementary Figure 2. Cell viability of integrin inhibitor cilengitide. The A549-WT cells were treated with different concentrations of Cilengitide for 24 h, and 100 μL of the CellTiter-Lumi™ Luminescence based Cell Vitality Detection regents (Beyotime #C0065M) was added to each well. After incubation for 10min at room temperature, luminescence was recorded by using a FlexStation 3 (Molecular Devices) with an integration time of 1 s per well.

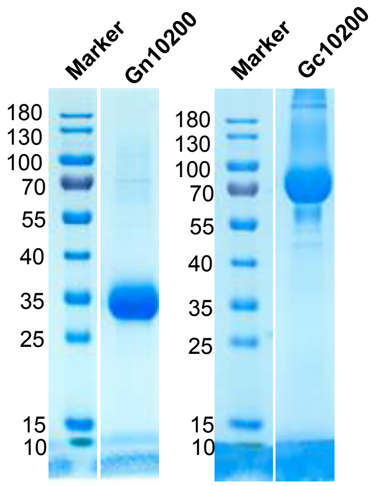

Supplementary Figure 3. The expression of Gn and Gc proteins of CCHFV IbAr10200 strain. The Gn and Gc of CCHFV IbAr10200 were expressed in Expi293F cells and purified using Strep-Tactin®XT 4Flow® resin. The eluted fractions were collected, concentrated, and subjected to SDS-PAGE, and analyzed by Coomassie staining.

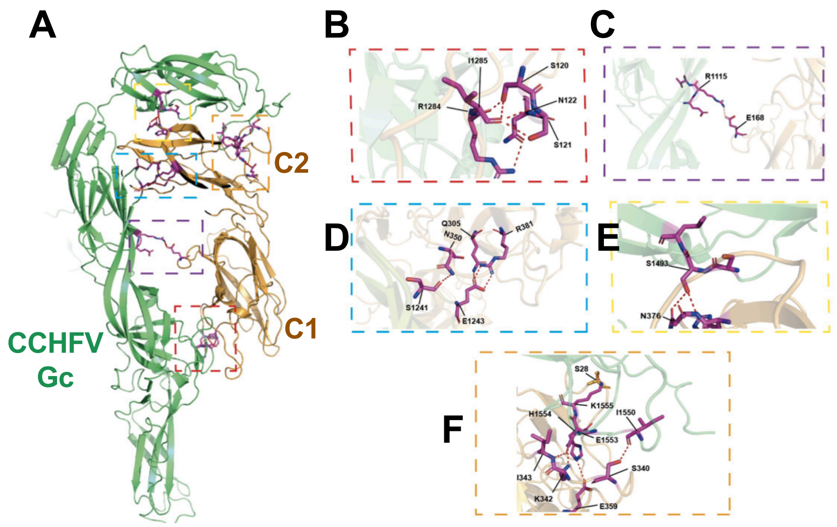

Supplementary Figure 4. In silico analysis of MFGE8 binding to CCHFV Gc protein. A. We employed AlphaFold3 to predict the structural interactions between Gc and MFGE8, and identified five binding interfaces. **B.** The first interface involves the C1 domain of MFGE8, where S120-S121-N122 interact with Gc R1284-I1285, forming hydrogen bonds between S120 and R1284/I1285, S121 and I1285, and N122 and

R1284. **C.** The second interaction site is between MFGE8 E168 (C1 domain) and Gc R1115. **D.** The third interface occurs between MFGE8 N350-Q305-R381 (C2 domain) and Gc S1241-E1243, with hydrogen bonds formed between Q305/R381 and E1243, and between N350 and S1241. **E.** The fourth binding site involves MFGE8 N376 (C2 domain) and Gc S1493. **F.** The fifth interface includes interactions between MFGE8 EGF/C2 domains and Gc, with S28 (EGF domain) forming a hydrogen bond with Gc K1555, and I343-K342-S340-E359 (C2 domain) interacting with Gc I1550-E1553-H1554.

SUPPLEMENTARY TABLE LEGENDS

Supplementary Table 1. List of genes and scores after MaGeck analysis of CRISPR knockout and activation screens (see Excel file).

Supplementary Table 2. List of primers and probes used for qRT-PCR experiments, and sgRNA sequences of genes selected for validation experiments (see Excel file).
